# Prospective Study of Dietary Zinc Intake and Risk of Cardiovascular Disease in Women

**DOI:** 10.3390/nu10010038

**Published:** 2018-01-04

**Authors:** Abul Hasnat Milton, Khanrin P. Vashum, Mark McEvoy, Sumaira Hussain, Patrick McElduff, Julie Byles, John Attia

**Affiliations:** Centre for Clinical Epidemiology and Biostatistics (CCEB), School of Medicine and Public Health, Faculty of Health and Medicine, The University of Newcastle, University Drive, Callaghan, NSW 2308, Australia; khanrin.vashum@newcastle.edu.au (K.P.V.); mark.mcevoy@newcatle.edu.au (M.M.); sumaira.87@gmail.com (S.H.); patrick.mcelduff@newcastle.edu.au (P.M.); julie.byles@newcastle.edu.au (J.B.); john.attia@newcastle.edu.au (J.A.)

**Keywords:** diet, cohort, Australia, zinc, women, cardiovascular disease

## Abstract

Several animal and human studies have shown that zinc is associated with cellular damage and cardiac dysfunction. This study aims to investigate dietary zinc and the zinc-iron ratio, as predictors of incident cardiovascular disease (CVD) in a large longitudinal study of mid-age Australian women (aged 50–61 years). Data was self-reported and validated food frequency questionnaires were used to assess dietary intake. Energy-adjusted zinc was ranked using quintiles and predictors of incident CVD were examined using stepwise logistic regression. After six years of follow-up, 320 incident CVD cases were established. A positive association between dietary zinc intake, zinc-iron ratio and risk of CVD was observed even after adjusting for potential dietary and non-dietary confounders. Compared to those with the lowest quintile of zinc, those in the highest quintile (Odds Ratio (OR) = 1.67, 95% Confidence Interval (CI) = 1.08–2.62) and zinc-iron ratio (OR = 1.72, 95% CI = 1.05–2.81) had almost twice the odds of developing CVD (*p* trend = 0.007). This study shows that high dietary zinc intake and zinc-iron ratio is associated with a greater incidence of CVD in women. Further studies are required detailing the source of zinc and iron in diet and their precise roles when compared to other essential nutrients.

## 1. Introduction

The prevalence of chronic diseases has been increasing in the past few decades. Cardiovascular diseases (CVDs) are the number one cause of death globally: more people die annually from CVDs than from any other cause [1]. CVD has a strong genetic basis and it is modified by environmental factors. To date, the most accessible method of preventing or lowering occurrence of CVD is through modification of lifestyle factors, especially diet and exercise. A review in 1996 by Houtman about trace elements and CVD obtained from epidemiological, biochemical and cell biological studies concluded that zinc has the potential to counteract the development of cardiovascular disease [2]; however, the strength of this effect on public health is difficult to measure. Recently, there has been growing interest in the relation of dietary intake, especially micronutrients present in the diet, and risk of CVDs [3,4,5,6]. Zinc is one of those micronutrients present in our diet whose deficiency may play an important role in the appearance of diseases and has three major biological roles, as catalyst, structural, and regulatory ion [4]. Zinc has been reported to have antioxidant and anti-atherosclerotic effects [4,5,7]. Zinc deficiency leads to cellular damage and atherosclerosis [5] and is known to cause sensitivity to oxidative damage, leading to an increased release of interleukin 1 and tumour necrosis factor-α, which causes increased endothelial cell apoptosis [7].

Experimental studies have shown that zinc administration during reperfusion improves myocardial recovery to almost 100%, protects against cardiac mechanical and/or electrical dysfunction, decreases the incidence of arrhythmias and improves post-ischemic myocardial recovery [8]. Another recent experimental study demonstrated that decreased level of zinc in the diet could induce hypertension and cardiac dysfunction [9]. Inadequate intake of zinc in humans was persistently observed in patients with heart disease [10] and trace element analysis of hair showed that CVD patients had lower concentrations of zinc [11]. Similarly, decreased serum zinc level was observed in ischemic stroke patients when compared to control patients [12].

Trials have explored the contribution of zinc supplementation in different conditions. In stroke patients, zinc supplementation has been shown to decrease the risk of mortality (Lixian trial) [13] and enhance neurological recovery [14]. However, according to a systematic review on the effect of zinc supplements in humans, besides the observed adverse effect on plasma high-density lipoprotein cholesterol (HDL-C) concentrations, the effect of zinc supplementation on heart disease risk remains unclear [15]. A more recent review of prospective cohort studies on zinc status and CVDs revealed no association between zinc intake and CVD events [6]. To the best of the authors’ knowledge, apart from the inconclusive findings in the aforementioned reviews [6,15], no prospective study has been carried out to investigate the association between dietary zinc intake and risk of incident CVD in women. Consequently, the objective of this study was to investigate whether dietary zinc was associated with incident cardiovascular disease in a large cohort of Australian women. A secondary aim was to examine the association of dietary zinc-iron ratio with CVD because minerals with similar physical or chemical properties such as iron and zinc may compete with each other biologically [16] and previous studies in humans have shown that iron interferes with the absorption of zinc [17].

## 2. Methods

### 2.1. The Australian Longitudinal Study on Women’s Health (ALSWH)

The Australian Longitudinal Study on Women’s Health (ALSWH) is a national cohort study examining the health and wellbeing of women of different ages. This paper uses data from the 1946–1951 cohort, who were 45–50 years of age during Survey 1 in 1996. Ethical approval was obtained by the Human Research Ethics Committees of the University of Newcastle (H-076-0795) prior to baseline data collection in 1996, with written informed consent provided by participants. Ethical clearance for ALSWH was obtained from the University of Newcastle and University of Queensland. Details of ALSWH’s methods and recruitment have been previously published [18,19]. Briefly, women were randomly selected from Medicare, the National Health Insurance Database, which includes all permanent residents of Australia. ALSWH collects self-reported data using mailed and online surveys at roughly 3-year intervals and has linked the study data with administrative records after the women gave their consent to participate in the study. The surveys include questions about: health conditions, symptoms, and diagnoses; use of health services; health-related quality of life; social circumstances, including work and time use; demographic factors; and health behaviors.

The response rate for the 1946–1951 cohort at Survey 3 in 2001 (then aged 50–55 years) was 83% of those who had completed Survey 1 (1996) and had not died (*n* = 115) or become too ill to complete further surveys (*n* = 21). Complete food frequency questionnaire (FFQ) data were available for 11,196 women aged 50–55 years (Survey 3, 2001) and 9264 of these women had data available for analysis when they were 56–61 years (Survey 5 in 2007).

### 2.2. Dietary Assessment

ALSWH uses the Dietary Questionnaire for Epidemiological Studies (DQES) Version 2 FFQ. Both the development of the questionnaire [20] and its validation in young Australian women has been previously reported [21]. This questionnaire asks respondents to report their usual consumption of 74 foods and six alcoholic beverages over the preceding 12 months using a 10-point frequency scale. Additional questions are asked about the number of serves or type of fruit, vegetables, bread, dairy products, eggs, fat spreads and sugar and further details are provided in Hodge et al. [21]. Nutrient intakes were computed from NUTTAB 1995, a national government food composition database of Australian foods NUTTAB 95 [22], using software developed by the Cancer Council of Victoria. The validation of the FFQ against a 7-day weighted food record showed Pearson correlation coefficient = 0.40 for dietary zinc and 0.44 for dietary iron. The FFQ validation study deemed the correlation coefficient acceptable as it is of similar magnitude to those previously reported [21].

### 2.3. Ascertainment of Cardiovascular Disease

History of CVD was collected at both baseline and follow-up. Participants were classified as having CVD if they had been diagnosed/treated by a doctor for heart attack and/or stoke which was self-reported. In this paper, incident cases of CVD at follow-up were classified as individuals with a diagnosis/treatment of heart attack and/or stroke, after excluding the prevalent cases; that is, those who self-reported a diagnosis/treatment of the condition at Survey 1, 2, or Survey 3.

### 2.4. Measurement of Non-Dietary Factors

Social and behavioral characteristics were based on information collected at Survey 3 in 2001. Participants were asked to report frequency of engaging in vigorous (e.g., aerobics, jogging) and less vigorous (e.g., walking and swimming) exercise lasting for <20 min in a normal week. Responses were scored using approximate weekly frequencies of exercise. The resulting physical activity scores ranged from 0 to 80 and were categorized as “nil/sedentary (<5)”, “low (5 to 15)”, “moderate (16 to 25)”, or “high (>25)”. A score of 15 is commensurate with the current recommendation of moderate intensity activity on most days of the week. This measure is described in more detail elsewhere [23] and has previously been shown to have acceptable test-retest reliability [24]. Standard questions were used to categorize respondents as never-smoker, ex-smoker, or current smoker; body mass index (BMI) was calculated as self-reported weight in kilograms, divided by height in meter squared. Medical history of hypertension and diabetes as diagnosed by a physician along with use of hormone replacement therapy (HRT) (coded as either yes or no) were all self-reported. The participants were also asked to report the number of supplements being used and categorized as taking multivitamin & mineral supplements (yes or no). Alcohol intake was quantified and classified according to Australian National Health & Medical Research (NHMRC, CBR, Australia) guidelines [25]. Annual household income and level of education was sub-classified into different categories.

### 2.5. Statistical Analysis

Zinc and iron were adjusted for total energy intake using the residual method [26]. The macronutrient variables are adjusted for total energy intake by calculating their component of total energy (as a %). The micronutrients are adjusted for total energy by regressing (using linear regression) the natural log of the micronutrient on the natural log of total energy and extracting the standardized residuals.

Associations between baseline characteristics and quintiles of energy-adjusted zinc were tested using chi-square for categorical variables and analysis of variance (ANOVA) for continuous variables. Quintiles of energy-adjusted zinc were obtained using the *xtile* command in Stata. Predictors of 6-year incidence of CVD were examined using forward stepwise multivariable logistic regression in stepped approach, with the main predictor being energy adjusted zinc and zinc-iron ratio measured at Survey 3 used to predict incidence of CVD by Survey 5.

The multivariable analysis controlled for dietary factors (energy adjusted fiber, fat and iron), non-dietary factors (BMI, smoking status, education, marital status, HRT, exercise group, history (yes/no) of hypertension and diabetes) along with alcohol intake and use of multivitamins/minerals. *p*-values for trends were conducted by treating quintile of energy-adjusted zinc as a continuous variable. Statistical significance was considered when 2-sided *p* < 0.05. STATA software version 11 was used for all statistical analyses.

## 3. Results

The baseline characteristics at Survey 3 of the participants by quintile of energy-adjusted dietary zinc (quintile 1 = lowest intake; quintile 5 = highest intake) are shown in Table 1. The dietary zinc intakes of the lowest and highest quintiles were 5.94 mg/day (95% Confidence Interval (CI) = 5.90, 5.99) and 17.35 mg/day (95% CI = 17.12, 17.59) respectively, with a mean intake of 10.66 mg/day. At baseline, (Table 1) non-dietary factors found to be significantly different across quintiles included: smoking status, exercise, education, household income, BMI, HRT, hypertension and age. Energy-adjusted dietary factors found both macronutrients and micronutrient to be significantly different across quintiles. Women in the highest quintile of zinc intake were more likely to be a current smoker and also have a higher level of alcohol intake. Interestingly, women with a higher intake of zinc also had a higher BMI whereas women in the lower quintiles were found to have a more sedentary lifestyle with little or no exercise. Mean age was similar across all the quintiles.

Table 2 and Table 3 show the output of the stepwise approach that examined energy adjusted zinc intake and zinc-iron ratio as independent predictors of incident CVD respectively. At the end of the six years follow-up, 320 (3.6%) incident cases of CVD were identified out of 9264 participants.

In an age adjusted analysis there was no significant association across quintiles 2, 3 and 4 but quintile 5 showed a significant association (OR = 1.43, 95% CI = 1.01–2.04) between energy-adjusted zinc and risk of CVD (Table 2). After adjustment for non-dietary factors including age, the findings did not change and quintile 5 remained statistically significant with *p* = 0.029 (OR = 1.44, 95% CI = 1.01–2.10). Further adjustment for dietary factors lead to an overall increase in odds of developing CVD and strengthened the association (*p* = 0.011). Additional adjustment for alcohol intake and use of supplements (multivitamins/minerals) also showed a statistically significant increase in the odds of developing CVD across quintiles (*P_trend_* = 0.007). Compared with the lowest quintile of energy-adjusted zinc those in the highest quintile had almost twice the odds of developing CVD (OR = 1.67, 95% CI = 1.08–0.77).

The association between the energy-adjusted zinc–iron ratio and odds of developing CVD (Table 3) was significant (*p* = 0.002) after adjusting for age with a consistent increase in the odds of developing CVD across the quintiles. A borderline statistically significant association was observed (*P_trend_* = 0.057) after adjusting for age and non-dietary factors. A statistically significant increase in the odds of developing CVD across quintiles of energy-adjusted zinc–iron ratio was observed with further adjustment for energy-adjusted dietary factors (*p* = 0.015) and alcohol intake and use of supplements (*p* = 0.007). Those in quintile 4 (OR = 1.78, 95% CI =1.13–2.79) and 5 (OR = 1.72, 95% CI = 1.05–2.81) had almost twice the odds of developing CVD compared with the lowest quintile of energy-adjusted zinc-iron ratio.

## 4. Discussion

Findings from this longitudinal study showed that dietary zinc intake was associated with a higher incidence of CVD in women aged 50 years and older, even after adjusting for potential dietary and non-dietary confounders. The same association was also observed between zinc-iron ratio and risk of CVD. To our knowledge, this is the only longitudinal study that has looked at the relationship between dietary zinc and zinc-iron ratio with incident CVD in an adult women population.

The association between dietary zinc and CVD suggests that high dietary zinc might increase the risk of CVD. The only other prospective study that looked at dietary intake of zinc and iron and CVD was the Iowa women’s health study [27]. The study found that dietary zinc and iron were not associated with risk of CVD mortality but the study might have lacked power. Furthermore, the end point of the study was CVD mortality and CVD incidence data was not measured; consequently, cases of CVD with a different cause of death may have been missed. A key observation noted was that the benefit of higher zinc intake in CVD mortality occurred only in the presence of a trigger such as alcohol (≥10 g/day) that could disturb iron homeostasis. Contrastingly, another study reported that alcohol consumption (>40 mL/day) was negatively linked to serum zinc [28]. Lee et al. 2005 also observed that women with higher dietary zinc intake were less likely to smoke and engaged in more physical activity [27], which differs from the characteristics observed in our study. It is widely known that smoking and BMI are modifiable risk factors for CVD and could be a possible explanation for the observed benefit of higher zinc intake in CVD mortality despite the presence of alcohol intake (≥10 g/day). The study also does not appear to have controlled for type 2 diabetes though it has considered other dietary and non-dietary factors as independent variables.

The relationship between type 2 diabetes and CVD is established: CVD events related to type 2 diabetes and the high incidence of other macrovascular complications are a major cause of chronic disease burden [29]. It has been shown that the age-adjusted prevalence of coronary heart disease is twice as high among those with type 2 diabetes than among those without diabetes [30,31]. Hence, zinc status in patients with type 2 diabetes and CVD events was assessed [32,33], which showed low serum zinc and high concentration of urinary zinc excretion in patients with diabetes mellitus and all CVD events [32,33]. Zinc intake was also assessed in an older population of men and women in Australia [34] and in the same cohort of women with type 2 diabetes [35]. These studies showed that a high zinc intake was inversely associated with risk of insulin resistance and type 2 diabetes.

In contrast to the observed association between type 2 diabetes and dietary zinc intake in the same cohort, the finding from this study indicates that dietary zinc intake after a certain level increases the risk of CVD and that those in the highest quintiles are above the threshold and was zinc replete. A possible explanation for this could be the fact that the participants are residents in Australia, a mainly affluent Western society, with reasonably good nutrition and were zinc replete. Of particular interest in this study, it was observed that those in the highest quintile of zinc also had the highest quintile of dietary iron intake. The most commonly recorded dietary source of zinc and iron was meat as the highest contributor, which could explain the increase in iron as zinc intake increases, though fish, poultry, cereals and dairy products were other substantial source. We cannot be sure whether the zinc intake is directly increasing the incidence of CVD, as zinc intake tends to increase as other micronutrients intakes increase (for example, iron in this study), or diet quality more generally. However, recent studies have shown that high intake of red meat in multivariable analyses including age, smoking, and other risk factors [36] and specifically, zinc and heme iron from red meat and not other sources [3] was associated with increased risk of CVD. Findings from these studies lend support to the association observed in our study, as the main source of zinc and iron in the majority of the cohort was most likely to be meat based on the Australian diet.

The secondary aim of this study was also to look at dietary zinc-iron ratio as part of the analysis because iron is known to interact with the absorption of zinc [16]. Zinc causes dose-dependent inhibition of iron binding to phospholipids and competition of zinc for iron binding sites is particularly relevant as zinc deficiency promotes intracellular iron accumulation [37]. We observed that risk of CVD was positively associated with higher quintiles of zinc-iron ratio. This suggests that the proportion of zinc intake in relation to iron is a relevant determinant of CVD risk. The association of zinc-iron ratio has also been observed in diabetes [35] in the same cohort even though the direction of association was different.

The main strengths of this study are the prospective design, where dietary assessment preceded the outcome, and the generalizability, this being a population-based cohort rather than a clinic sample. The main advantage of the prospective design is that it reduces selection bias and potential recall bias. It is also the only prospective study so far that looks at incident CVD with both dietary zinc and zinc-iron ratio. The large sample size also means that it is possible to obtain reasonably stable parameter estimates.

Despite the good generalizability of this study there are some limitations. Use of FFQ to collect dietary information (especially micronutrients) has limitations due to the lack of homogeneity in food composition tables [38] and over or under reporting of certain foods/food groups. However, the use of FFQs to collect dietary information in large population-based samples is the most cost-effective and feasible method available. In this study the use of an FFQ to estimate dietary zinc intake will have underestimated the amount of zinc consumed by study participants and biased the effect size towards the null [39]. A further limitation is that dietary assessment was carried out at one time only. However, instead of excluding participants who developed comorbidities during the follow-up period, we controlled for these in the analysis so as to account for any change in dietary habit that may have occurred following the development of comorbid conditions. Adjustments were made for all known major confounders but residual confounding cannot be entirely excluded. Also despite controlling for type 2 diabetes and alcohol intake, stratifying analysis by alcohol status to duplicate findings by Lee et al. 2005 [27] will be worthwhile in the future.

This study also considered the use of supplementary multivitamins/minerals and analysis showed that the proportion of people taking supplements were not significantly different between quintiles of zinc intake. Although multiple dietary assessments are useful to reduce random measurement error, given that single dietary assessment is likely to bias results toward the null, the fact that we still observed a significant association is an indication that the association is likely to be robust and even stronger than that observed in this study.

In conclusion, this prospective study showed that higher total dietary zinc intake and zinc–iron ratio is associated with a higher incidence of CVD in women aged 50 and above. This study finding is preliminary and needs to be investigated further. Future research is necessary to confirm this association in both men and women across different age groups. A positive finding should prompt further research to investigate the association of zinc and iron from meat and other major sources because these dietary aspects would encourage change in dietary guidelines recommended for reducing CVD burden.

## Figures and Tables

**Table 1:** Characteristics of subjects at baseline (Survey 3) by quintile of energy-adjusted zinc intake.

		Quintile of Energy-Adjusted Zinc Intake			
Characteristic	Sub-Group/Mean (SD)	Q1 Lowest (*n* = 2125)	Q3 Middle (*n* = 2125)	Q5 Highest (*n* = 2124)	*p*-Value
Smoking status	Never a smoker	948 (* 53%)	1033 (58%)	922 (52%)	<0.001
	Former smoker	568 (32%)	531 (30%)	569 (32%)	
	Current smoker	261 (15%)	215 (12%)	287 (16%)	
Alcohol Intake	Abstainer/rarely drinks	960 (49.84%)	762 (39.08%)	748 (37.72%)	<0.001
	Low risk/moderate drinker	902 (46.83%)	1088 (55.79%)	1049 (52.90%)	
	Binge/risky drinker	53 (2.75%)	93 (4.77%)	156 (7.87%)	
	Chronic/High risk drinker	11 (0.57%)	7 (0.36%)	30 (1.51%)	
Exercise group	Nil/sedentary	321 (19%)	271 (16%)	323 (19%)	0.003
	Low	621 (37%)	683 (40%)	622 (36%)	
	Moderate	318 (19%)	355 (21%)	338 (20%)	
	High	437 (26%)	408 (24%)	433 (25%)	
Education	No formal qualification	417 (19.80%)	300 (14.23%)	365 (17.29%)	<0.001
	School/intermediate certificate	597 (28.35%)	658 (31.21%)	764 (36.19%)	
	Secondary schooling completed	343 (16.29%)	372 (17.65%)	342 (16.20%)	
	Trade qualification/TAFE	415 (19.71)	435 (20.64%)	397 (18.81%)	
	University/other tertiary study	334 (15.86%)	343 (16.27%)	243 (11.51%)	
Annual household income	$1–$15,999	127 (9.25%)	82 (5.27%)	86 (5.66%)	<0.001
	$16,000–$36,999	415 (30.23%)	398 (25.56%)	428 (28.18%)	
	$37,000–$77,999	472 (34.38%)	589 (37.83%)	545 (35.88%)	
	$78,000 or more	189 (13.77%)	276 (17.73%)	253 (16.66%)	
	Don’t know or missing	170 (12.38%)	212 (13.62%)	2017 (13.63%)	
Hormone Replacement Therapy (HRT)	No	1250 (70%)	1193 (67%)	1160 (65%)	0.020
	Yes	535 (30%)	591 (33%)	624 (35%)	
Hypertension	No	1498 (85%)	1495 (85%)	1449 (82%)	0.038
	Yes	271 (15%)	274 (15%)	316 (18%)	
Diabetes	No	1724 (99.60%)	1790 (99.50%)	1719 (99.42%)	0.659
	Yes	7 (0.40%)	9 (0.50%)	10 (0.58%)	
Use of multivitamins/minerals	No	957 (45.38%)	918 (43.47%)	989 (46.78%)	0.260
	Yes	1152 (54.62%)	1194 (56.53%)	1125 (53.22%)	
Age	mean (SD)	52.6 (1.4)	52.6 (1.5)	52.4 (1.5)	0.004
Body Mass Index	mean (SD)	26.0 (5.4)	26.5 (5.1)	27.2 (5.4)	<0.001
Total energy intake (KJ/day)	mean (SD)	6676 (2415)	6604 (2275)	6687 (2790)	0.508
Carbohydrates (% of energy)	mean (SD)	48.2 (5.8)	45.6 (5.8)	41.0 (7.5)	<0.001
Dietary fibre (% of energy)	mean (SD)	2.4 (0.7)	2.5 (0.6)	2.5 (0.8)	<0.001
Total protein (% of energy)	mean (SD)	17.1 (2.1)	20.8 (1.7)	25.1 (2.8)	<0.001
Total fat (% of energy)	mean (SD)	35.5 (5.5)	34.3 (5.9)	34.5 (6.6)	<0.001
Saturated fat (energy adjusted)	mean (SD)	14.0 (3.8)	13.5 (3.4)	13.9 (3.3)	<0.001
Polyunsaturated fat (% of energy)	mean (SD)	6.3 (2.3)	5.5 (1.9)	4.7 (1.5)	<0.001
Monounsaturated fat (% of energy)	mean (SD)	12.1 (2.2)	12.1 (2.4)	12.6 (2.8)	<0.001
Iron mg/day (energy adjusted)	mean (SD)	−0.605 (0.984)	0.022 (0.905)	0.543 (0.897)	<0.001
Cholesterol mg/day (energy adjusted)	mean (SD)	−0.584 (1.170)	−0.043 (0.827)	0.607 (0.855)	<0.001
Retinol ug/day (energy adjusted)	mean (SD)	0.451 (0.947)	0.030 (0.932)	−0.525 (0.993)	<0.001
Vitamin C mg/day (energy adjusted)	mean (SD)	−0.092 (1.198)	0.024 (0.957)	0.028 (0.895)	0.001
Vitamin E mg/day (energy adjusted)	mean (SD)	0.346 (1.094)	0.035 (0.923)	−0.407 (0.939)	<0.001
Calcium mg/day (energy adjusted)	mean (SD)	−0.374 (0.901)	0.126 (0.940)	0.089 (1.166)	<0.001
Magnesium mg/day (energy adjusted)	mean (SD)	−0.453 (1.001)	0.064 (0.901)	0.268 (1.069)	<0.001
Sodium mg/day (energy adjusted)	mean (SD)	−0.432 (1.030)	0.026 (0.930)	0.345 (0.999)	<0.001
Potassium mg/day (energy adjusted)	mean (SD)	−0.594 (1.072)	0.067 (0.891)	0.454 (0.915)	<0.001

* The percentage (%) for each characteristic in the quintile columns is % of the total. *p*-Values are for trends.

**Table 2:** Stepwise multivariable logistic regression to examine energy-adjusted zinc intake as an independent predictor of a new diagnosis of cardiovascular disease.

	Quintile of Energy-Adjusted Zinc Intake					
	Q1	Q2	Q3	Q4	Q5	* *p*
Energy-adjusted zinc (median (min, max))	−1.25 (−4.8, −0.79)	−0.48 (−0.79, −0.23)	0.01 (−0.23, 0.26)	0.50 (0.26, 0.79)	1.24 (0.79, 4.45)	
Number of cardiovascular disease	54	55	62	72	77	
Odds ratio						
Age adjusted	1.00	0.99 (0.68 to 1.46)	1.10 (0.76 to 1.60)	1.28 (0.90 to 1.84)	1.43 (1.01 to 2.04)	0.015
Age & non-dietary ^†^ factors adjusted	1.00	0.92 (0.63 to 1.48)	1.19 (0.84 to 1.88)	1.16 (0.82 to 1.83)	1.44 (1.01 to 2.10)	0.029
Age, non-dietary ^†^ and dietary ^‡^ factors adjusted	1.00	0.96 (0.62 to 1.47)	1.26 (0.84 to 1.90)	1.25 (0.82 to 1.90)	1.63 (1.07 to 1.90)	0.011
Age, non-dietary ^†^ and dietary ^‡^ factors adjusted plus alcohol intake and use of supplements	1.00	0.94 (0.60 to 1.48)	1.18 (0.76 to 1.82)	1.36 (0.84 to 2.00)	1.67 (1.08 to 2.62)	0.007

^†^ Non-dietary factors were BMI; smoking status; HRT; exercise group; education level and history of diabetes and hypertension; ^‡^ Dietary factors were energy-adjusted fiber, iron and fat. Adjustment for family income in the models resulted in a loss of 1300 observations but education was adjusted for. * *p*-Value for trends.

**Table 3:** Stepwise multivariable logistic regression to examine zinc–iron ratio as an independent predictor of a new diagnosis of cardiovascular disease.

	Quintile of Zinc-Iron Ratio					
	Q1	Q2	Q3	Q4	Q5	* *p*
Zinc-iron ratio (median(min, max))	0.69 (0.28, 0.77)	0.84 (0.77, 0.90)	0.95 (0.90, 1.00)	1.06 (1.00, 1.12)	1.21 (1.12, 1.75)	
Number of cardiovascular disease	51	52	69	74	74	
Odds ratio						
Age adjusted	1	1.02 (0.69 to 1.51)	1.38 (0.96 to 1.99)	1.51 (1.05 to 2.17)	1.55 (1.08 to 2.23)	0.002
Age & non-dietary ^†^ factors adjusted	1	0.95 (0.61 to 1.47)	1.38 (0.92 to 2.06)	1.47 (0.99 to 2.09)	1.40 (0.93 to 2.09)	0.057
Age, non-dietary ^†^ and dietary ^‡^ factors adjusted	1	1.03 (0.66 to 1.59)	1.45 (0.95 to 2.20)	1.57 (1.02 to 2.42)	1.54 (0.97 to 2.45)	0.015
Age, non-dietary ^†^ and dietary ^‡^ factors adjusted plus alcohol intake and use of supplements	1	1.15 (0.73 to 1.83)	1.52 (0.97 to 2.36)	1.78 (1.13 to 2.79)	1.72 (1.05 to 2.81)	0.007

^†^ Non-dietary factors were BMI; smoking status; HRT; exercise group; education level and history of diabetes and hypertension. ^‡^ Dietary factors were energy-adjusted fiber and fat. * *p*-Value for trends.
